# Atrous residual convolutional neural network based on U-Net for retinal vessel segmentation

**DOI:** 10.1371/journal.pone.0273318

**Published:** 2022-08-22

**Authors:** Jin Wu, Yong Liu, Yuanpei Zhu, Zun Li

**Affiliations:** 1 School of Information Science and Engineering, Wuhan University of Science and Technology, Wuhan, China; 2 School of Medical Engineering, Xinxiang Medical University, Xinxiang, China; 3 School of Physics and Electronic Engineering, Xinxiang University, Xinxiang, China; Politechnika Slaska, POLAND

## Abstract

Extracting features of retinal vessels from fundus images plays an essential role in computer-aided diagnosis of diseases, such as diabetes, hypertension, and cerebrovascular diseases. Although a number of deep learning-based methods have been used in this field, the accuracy of retinal vessel segmentation remains challenging due to limited densely annotated data, inter-vessel differences, and structured prediction problems, especially in areas of small blood vessels and the optic disk. In this paper, we propose an ARN model with a atrous block to address these issues, which can avoid the loss of data structure, and enlarge the receptive field, so that each convolution output contains a larger range of information. In addition, we also introduce residual convolution network to increase the network depth and improve the network performance.Some key parameters are used to measure the feasibility of the model, such as sensitivity (Se), specificity (Sp), F1-score (F1), accuracy (Acc), and area under each curve (AUC). Experimental results on two benchmark datasets demonstrate the effectiveness of the proposed methods, which accuracy are 0.9686 on the DRIVE and 0.9746 on the CHASE DB1. The segmentation structure can assist the doctor in diagnosis more effectively.

## Introduction

Diseases such as diabetes, hypertension, and diseases of the retina are shown in retinal vascular images [1]. The analysis of the number, angle, branch, and curvature of retinal blood vessels can provide a valuable basis for clinical diagnosis [2], for the purpose of early prevention, diagnosis, and treatment. With the development and popularity of optical coherence tomography (OCT) imaging technology and the increasing emphasis on early disease diagnosis, the number of fundus images is increasing rapidly, and their analysis will require much time and effort [3]. In addition, differences in image acquisition procedures between machines and institutions may lead to huge differences in resolution, noise, and tissue appearance, which increase the difficulty of analysis [4].

To meet the need of this work, a fast and automatic segmentation method for retinal vascular images came into being [5,6]. It can improve the cutting efficiency and accuracy, reduce the waiting time of patients and save medical resources [7,8]. It can also provide basis for fundus image registration, arteriovenous classification and biometric recognition [9]. Notably, deep learning methods have performed better than traditional methods [10–13].

Among the unsupervised methods, filtering, morphological transformation and model-based algorithms are dominant [14]. The method was evaluated on DRIVE and STARE databases and returned accuracies of 0.945 and 0.9486 respectively.Wavelet transform was used by Akram et al. and Soares et al. in retinal vascular segmentation [15], and they achieved 94.4% accuracy in STARE. 2D Gabor Wavelet and Gaussian mixture models are used in their approach. In particular, this approach is heavily influenced by the quality of the image. The entropy of some particular antennas with a pre-fractal shape, harmonic sierpinski gasket and weierstrass-mandelbrot fractal function were studied, and the result indicated that their entropy is linked with the fractal geometrical shape and physical performance [16,17], and they achieved 94.3% and 94.4% accuracy in DRIVE and STARE. This method is a kind of unsupervised technique, and the calculation is fast, but the accuracy is limited. Frangi et al. proposed a multi-scale enhanced-vessel filtering method to enhance vascular and vascular-like patterns, in which second-order local structural features were used [18]. Sato et al. applied three-dimensional (3D) multi-scale line filtering to the segmentation of cerebrovascular, bronchial and liver vessels [19]. This method can improve the continuity of the circuit structure and reduce the noise, but the calculation is large and slow. Jiang et al. proposed a universal vessel segmentation framework based on adaptive local threshold and applied it to retinal vessel segmentation [20], they observed 65% true positive rate, and This method has more parameters and slower operation. Zhang et al. proposed a filter based segmentation method for retinal vessels, which uses a locally adaptive derivative filter [21], and they achieved 94.76% and 95.54% accuracy in DRIVE and STARE. Azzopardi et al. improved COSFIRE operator detection and applied it in retinal segmentation [22], and they achieved 94.27% and 94.11% accuracy in DRIVE and CHASE_DB1. Zhao et al. designed a new retinal vessel segmentation model using an infinite parameter active contour model with mixed regional information [23], and they achieved 95.4% and 95.6% accuracy in DRIVE and STARE. Liang et al. proposed a level set method for vessel segmentation based on regional energy fitting information and shape prior probability [24] and they achieved 95.03% and 95.36% accuracy in DRIVE and STARE. The framework of unsupervised segmentation method always uses the filtering method which is sensitive to blood vessels or vessel-like, which will lead to the incomplete blood vessels and the misidentification of vessel-like parts. Moreover, the parameter setting has great influence on the final segmentation result.

For the supervised methods, ground truth must be used to train the classifier, and then the classifier can be used to extract the blood vessels. The features of retinal blood vessels can be extracted by multiple methods [25,26]. Traditional machine learning methods for training classifiers use k nearest neighbors, adaboost, random forest and other methods [27], and they achieved 92.9% accuracy in DRIVE. Orlando et al. proposed a fully connected conditional random field model for retinal vascular segmentation, using structured output support vector machine learning model parameters as an example to improve the effect [28], and they achieved 0.8741 and 0.8628 G-mean value in DRIVE and STARE. Zhang et al. applied retinal vascular segmentation through filtering and wavelet transform strategy, and used random forest training strategy [29], and they achieved 94.66% and 95.47% accuracy in DRIVE and STARE. In the above methods, the key feature selection has a great influence on the final segmentation result, such as whether features are independent or easy to identify. However, these features must be selected through people’s experience, The features need to be selected manually according to the experiment; so there is still much work to be done to perfect their shortcomings.

Benefiting from the rapid development of computer hardware, convolutional neural networks (CNNs) [30] have become the main machine learning method. Many CNN-based classification and detection methods have been proposed, which have facilitated the rapid development of medical assisted diagnosis methods. In the process of retinal vascular segmentation, the proportion of vascular areas, especially capillaries, is relatively small. To achieve a better segmentation effect, it is necessary to increase the number of training sets and the amount of training time. However, the number of training sets is limited in existing public datasets. To solve this problem, Ronneberger et al. proposed U-Net [31], which combines coarse and fine features through skip connections, can achieve better accuracy(95.34% and 95.78% accuracy in DRIVE and STARE) with fewer training sets. Many methods based on U-Net have achieved good results, but there are still problems such as low accuracy, poor sensitivity, and segmentation area error, especially the loss of vessel branch points, intersection points, and small vessels.The results by other authors are summarized in Table 1.

**Table 1:** Summary of related work.

Method	Sensitivity	Specificity	Accuracy
Mo and Zhang	0.8147	0.9844	0.9674
Neto et al.	0.8344	0.9443	0.8894
Kamble et al.	0.7177	0.9664	0.9421
Nugroho et al.	0.8927	0.7852	0.9022
Strisciuglio et al.	0.7716	0.9701	0.9497
Bahadar Khan etal.	0.758	0.9627	0.9458
Singh and Srivastava	0.7939	0.9376	0.9270
Zhao et al.	0.78	0.978	0.9560
Azzopardi et al.	0.7716	0.9701	0.9497
Nergiz et al.	0.8126	0.9442	0.9312
Mendonça	0.6996	0.027	0.9440
Staal	0.697	0.019	0.9516
Soares	0.7165	0.0252	0.9480
Singh and Srivastava	0.6134	0.0245	0.9384
Martinez-Perez	0.7506	0.0431	0.941
MF-FDOG	0.7177	0.0247	0.9484
Abdallah	0.6145	0.0162	0.9402
Raja et al.	0.936	0.9896	0.9594
Xiao et al.	0.7147	0.9735	0.9476
Manoj et al.	0.9314	0.9884	0.9583
Marín et al.	0.6944	0.9819	0.9526
Soares et al.	0.7181	0.9765	0.9500
Li et al.	0.7843	0.9837	0.9690

We propose ARU-Net, a deep learning model to automatically segment retinal blood vessels in fundus images. The model leverages the strengths of U-Net, cascaded atrous convolution, and residual blocks enriched with squeeze and excitation. Residual blocks [32] are used as building units to simplify the training process and help extract coarse and fine features from source images. Squeeze and excitation units are added to each remaining block for channel attention, adaptive feature recalibration, and increased feature power representation. The addition of a dilated convolution module can ensure global and multi-scale extraction. We evaluated our model on the publicly available DRIVE [33] and CHASE DB1 [34] datasets, and the results show that it is effective, and the performance is improved.

The proposed approach has the following contributions.

1. We propose a U-Net model integrating modified residual blocks to improve network performance.
2. An improved hybrid atrous convolution is used to increase the receptive field.

The rest of this paper is organized as follows: Section 2 analyze relevant literature. Section 3 presents the proposed method; Section 4 analyzes and discusses the experiment result; Section 5 concludes this study.

## Related work

Due to the excellent performance of deep learning framework in retinal vascular segmentation, We will analyze ralated works using typical deep learning architectures.

### U-Net

U-Net can be divided into three parts: left (down-sampling), middle (copy and crop), and right (up-sampling). The first part reduces the size of the picture through four down-sampling operations, which extract features from shallow information. The copy and crop part includes four splicing operations. This operation fuses characteristic deep and shallow information. In the up-sampling part, the picture is larger, and deep information is extracted through four up-sampling operations. In the process of up-sampling, the number of channels in the image is halved, which is contrary to the change of the number of channels in feature extraction in the left part [35]. The up-sampling process fuses the shallow information on the left and splices the features. A skip connection is used in U-Net at the same stage, ensuring that the recovered feature graph integrates more low-level features and features of different scales. In this way, multi-scale prediction and deep supervision can be carried out, and information such as edge recovery of segmentation maps can be more refined [36].

### ResNet

It has been found that deeper network layers and smaller receptive fields can improve neural network performance. However, as the network structure deepens, two problems arise. First, vanishing and exploding gradients affect the convergence of training. Second is degradation. An increasing number of layers causes the model accuracy to decrease (which is not caused by overfitting), and the training and testing error both increase.To overcome these problems, the The residual network proposed by He et al. [37] shows significantly improved training characteristics, allowing previously unachievable network depths.

In contrast to the traditional convolutional or fully connected layer, ResNet has many bypass branches that connect the input directly to the following layer, so as to directly learn the residuals. This structure is also known as a shortcut connection. Such a structure can directly detour the input information to the output to protect its integrity. The network only needs to learn the input and output differences of that part, simplifying the learning objectives and decreasing difficulty.

## Method

Using U-Net as the basic framework in medical image segmentation can solve the problem of small samples that commonly exist in such images. However, U-Net is composed of a contraction path that gradually reduces the spatial dimension of the image through down-sampling, and an expansion path that gradually restores the details and spatial dimension of the object through up-sampling. Therefore, after convolution and down-sampling, there will be gradient disappearance, structural information loss, and other problems.

**Fig 1:**
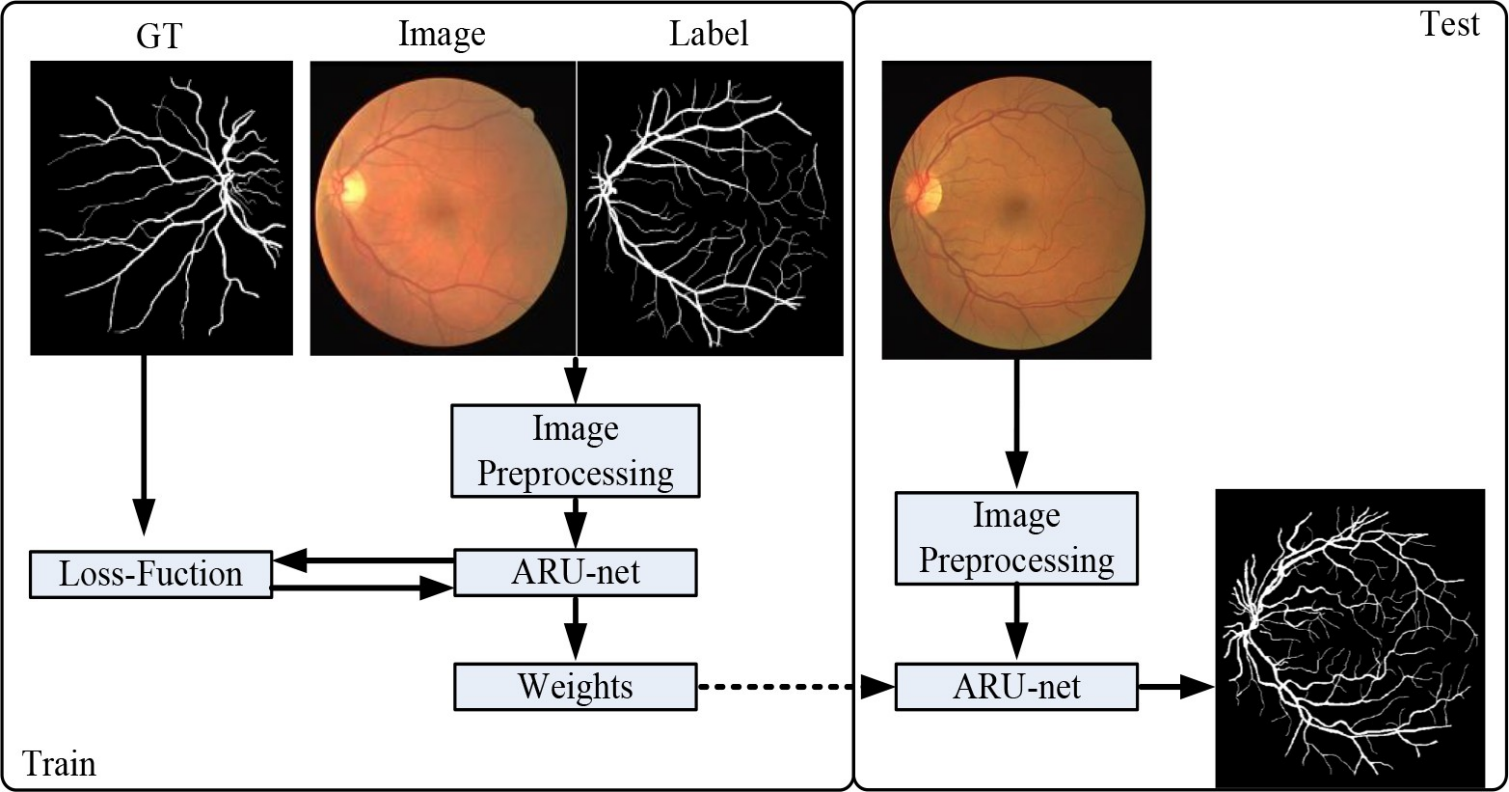
Overview of proposed ARU–Net.

In this study, We integrate a residual network (ResNet) and atrous convolution modules into the U-Net network in a new network structure, the atrous residual U-Net (ARU-Net), which can further expand the receptive field and improve the correlation between objects without losing information, thus improving the performance of vascular segmentation.This framework is shown in Fig 1. It consists of two phases of training and testing. In the training stage, color fundus images were preprocessed with grayscale transformation and normalization, and then used as training data. The network adjusts model weight parameters by iterative learning. Then save the weights. In the testing stage, the network reloads the saved weight information and makes predictions for the preprocessed data.

### Modified residual block

**Fig 2:**
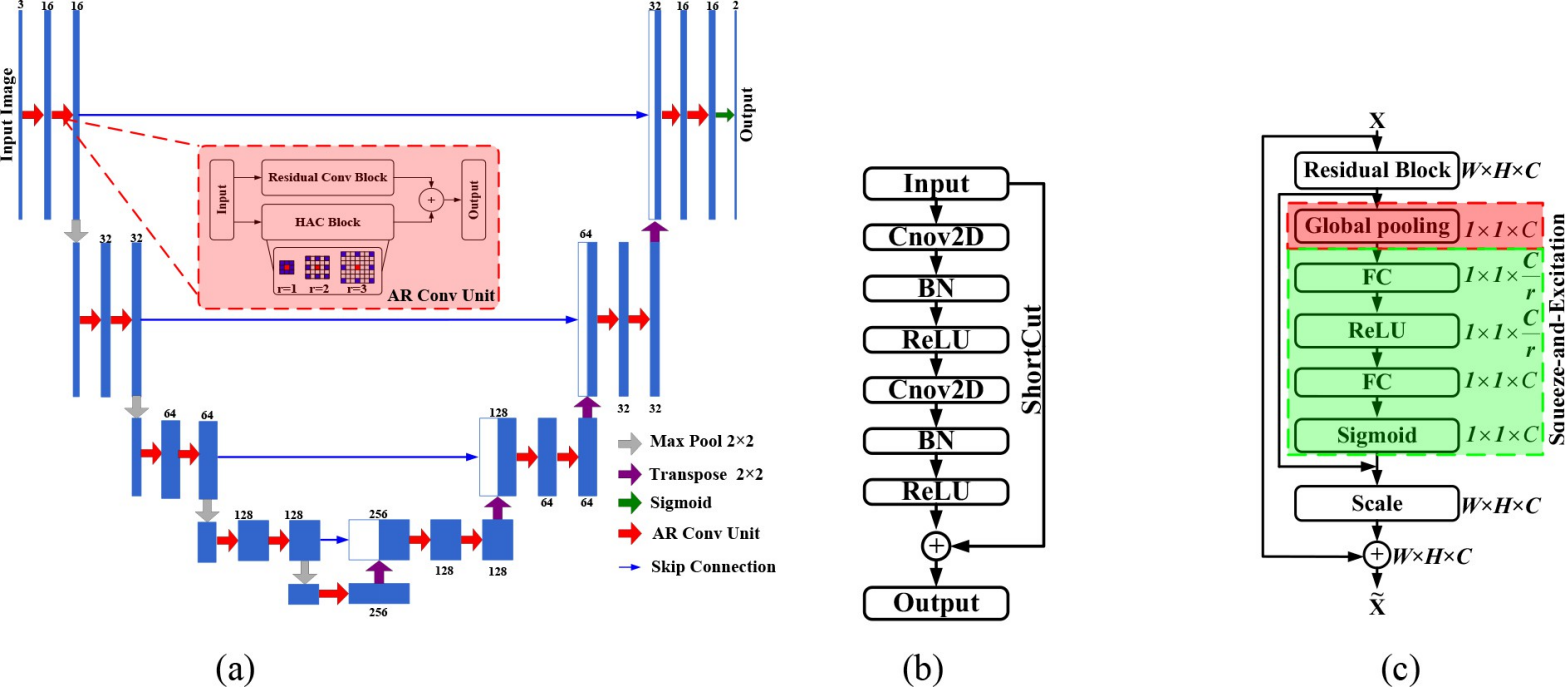
(a) Proposed ARU–Net Block, (b) standard residual block, (c) modified residual block.

To further improve the performance of the network, we include a squeeze-and-excitation block in ResNet [38], the difference from the original residual network is shown in Fig 2B and 2C. Squeeze (red box, Fig 2(C)) can change the spatial dimension of each input feature map from *H*×*W* squeeze to ***1***×***1***. We use global average pooling to achieve this. In the squeeze operation, we perform feature compression along the spatial dimension, turning each two-dimensional feature channel into a real number that has a global receptive field, and the dimension of the output matches the number of feature channels of the input. It represents the global distribution of responses on the characteristic channel and enables the layer close to the input to obtain the global receptive field, which is useful in many tasks.


$$z_{c}=F_{sq}(u_{c})=\frac{1}{H\times W}\sum_{i=1}^{H}\sum_{j=1}^{W}u_{c}(i,j)$$ (1)

Where *z*_*c*_ represents the channel descriptor for channel c, *F*_*sq*_ represents global average pooling, *u*_*c*_ represents channel c of the input, and *H* and *W* represent the height and width of the input.

In excitation (green box, Fig 2(C)), the feature dimension is reduced to 1/r of the input, and raised back to the original dimension through a fully connected layer after ReLU activation. This method, which is more nonlinear and can better fit the complex correlation between channels than the method that directly uses a fully connected layer, greatly reduces the number of parameters and the amount of calculation.

### Hybrid atrous convolution block

As is well known, atrous convolution can enlarge the receptive field [39,40]. When the convolution kernel is ***3***×***3***, 1-dilated and 2-dilated together can achieve the effect of a *7*×*7* convolution kernel. Similarly, when 4-dilated conv is followed by 1-dilated and 2-dilated conv, the receptive field can achieve the effect of a ***15***×***15*** convolution kernel. Compared with traditional convolution operations, the receptive field of atrous convolution grows exponentially.

However, when we only stack convolution with the same void rate many times, the kernel is not continuous, i.e., not all pixels are used for calculation. Therefore, the information is regarded as checkerboard, which will lose continuity, and does not work well for small objects.

We use a hybrid atrous convolution block to solve this problem,

$$M_{i}=\max[M_{i+1}-2r_{i},M_{i+1}-2(M_{i+1}-r_{i}),r_{i}],$$ (2)

where *r*_*i*_ is the void rate of the i-th layer, and *M*_*i*_ is the maximum void rate at layer i. Assuming there are n layers, the default is *M*_***n***_ = *r*_*n*_. If we apply a *k*×*k* convolution kernel and our goal is that *M*_2_≤*K*, then we can cover all the holes using standard convolution.

Proposed U-net Block is shown in the Fig 2A. We replaced the original COV3×3 block in the original U-net with AR Conv Unit. The red arrow represents AR Conv Unit, it is also an important difference from U-Net, and we can see the specific algorithm flow from Algorithm 1.

## Algorithm 1: Algorithm of the proposed ARU convolution unit

    **Input:** Feature map X

    **Output:** Feature map Y

    1: X_1_ = **HAC**(X) // Hybrid atrous convolution block with r[1,2,3]

    5: X_2_ = **h**(X)+F(x,w);xl+1 = f(x2)

    6: X_3_ = **F**_**tr**_(X2) // F_tr_: X→U, *X*∈*R*^*W*’×*H*’×*C*’^, *U*∈*R*^*W*×*H*×*C*^

    7: X_4_ = **F**_**Sq**_(X_3_) //$F_{Sq}(X)=\frac{1}{H\times W}\sum_{i}^{H}\sum_{j}^{W}X(i,j)$ Shrinking feature maps∈*R*^*W*×*H*×*C*^ through saptical dimentions

    8: X_5_ = F_ex_(X_4_,W) //Learnning *W*∈*R*^*C*×*C*^ to explicitly model channel-association

    9: X_6_ = F_scale_(X_2_,X_5_) //Reweighting the feature maps∈*R*^*W*×*H*×*C*^

    10: Y = concatenate(X_1_,X_6_)

## Experiments

### Implementation details

We evaluated the proposed ARU-Net on the DRIVE and CHASE DB1 retinal image datasets. DRIVE includes 40 color fundus images, with 20 for training and 20 for testing. CHASE DB1 contains 28 retinal fundus images. Although there is no initial division of training and testing sets, the first 20 are usually used for training, and the remaining eight for testing. Additionally, in Chase DB1, there are two sets of Ground-truth (GT) images, we chose the first group because they have more complete vessel details than broken ones. Images in DRIVE and CHASE DB1 have resolution of 565×584 and 999 × 960, respectively. To fit our network, we resize each image in DRIVE and CHASE DB1 to 592 × 592 and 1008 × 1008 by padding it with zero in four margins, but in evaluation, we crop the segmentation results to the initial resolution. Manually segmented binary vessel maps of both datasets provided by human experts can be applied as the ground truth.

The experiments have been conducted in a desktop computer with intel core i5-9400 processor CPU, 32GB RAM, and NVIDIA 1080Ti, 11 GB GPU. Adam optimization method was used to optimize the parameters. Since the phased training can speed up the convergence of the network, we adopted different parameters in the training process [41]. When using the Drive data set, we find that the first 200 epochs use learning rate of 0.003, followed by the learning rate of 0.0001 can achieve good results. And the same is true for CHASE DB1. The difference is that we set the batch size to be 2 and 1 respectively. The relevant model dimensions are listed in Table 2.

**Table 2:** Related parameters to our model and U–net.

Model	Parameters	Flops
U-Net[32,64,128]	517090	1032931
Proposed[16,32,64,128,256]	1054523	2100906

### Evaluation metrics

To quantitatively evaluate our model, we compared the segmentation results with the corresponding ground truth; divided the results of each pixel into true positive (TP), false positive (FP), false negative (FN), and true negative (TN); and adopted sensitivity (Se), specificity (Sp), F1-score (F1), and accuracy (ACC) to evaluate the performance of the model.


$$Se=\frac{TP}{TP+FN},$$ (3)

$$Sp=\frac{TN}{TN+FP},$$ (4)

$${\mathrm{precision}}_{K}=\frac{TP}{TP+FP},$$ (5)

$${\mathrm{recall}}_{K}=\frac{TP}{TP+FN},$$ (6)

$$F1={\left(\frac{1}{n}\sum \frac{2\cdot {\mathrm{precision}}_{K}\cdot {\mathrm{recall}}_{K}}{{\mathrm{precision}}_{K}+{\mathrm{recall}}_{K}}\right)}^{2},$$ (7)

$$A\mathrm{cc}=\frac{TP+TN}{TP+FN+TN+FP}.$$ (8)

We also utilize the area under the ROC curve (AUC), where a value of 1 indicates perfect segmentation.

## Results

The results in Table 3 are our results on public datasets, the DRIVE and the CHASE DB1. We compare the single use of residual networks, the single use of empty convolution and our approach. When the residual convolution block or the hybrid atrous convolution block is added separately, the segmentation performance of the network can be improved. The experimental results show that when the above two kinds of convolution are added to the network, the segmentation network can get better results.

**Table 3:** Experimental result on DRIVE and CHASE DB1.

Datasets	DRIVE					CHASE DB1				
**Architecture**	**Se**	**Sp**	**F1**	**ACC**	**AUC**	**Se**	**Sp**	**F1**	**ACC**	**AUC**
U-Net	0.7537	0.9820	0.8142	0.9531	0.9755	0.8288	0.8288	0.7783	0.9578	0.9578
RE only	0.7726	0.7726	0.8149	0.9553	0.9553	0.7726	0.7726	0.7800	0.9553	0.9553
Atrous only	**0.8130**	0.9819	**0.8223**	0.9680	0.9827	**0.8300**	0.9848	**0.8073**	0.9732	0.9860
Ours	0.8043	**0.9844**	0.8179	**0.9686**	**0.9842**	0.8099	**0.9856**	0.8005	**0.9746**	**0.9869**

**Fig 3:**
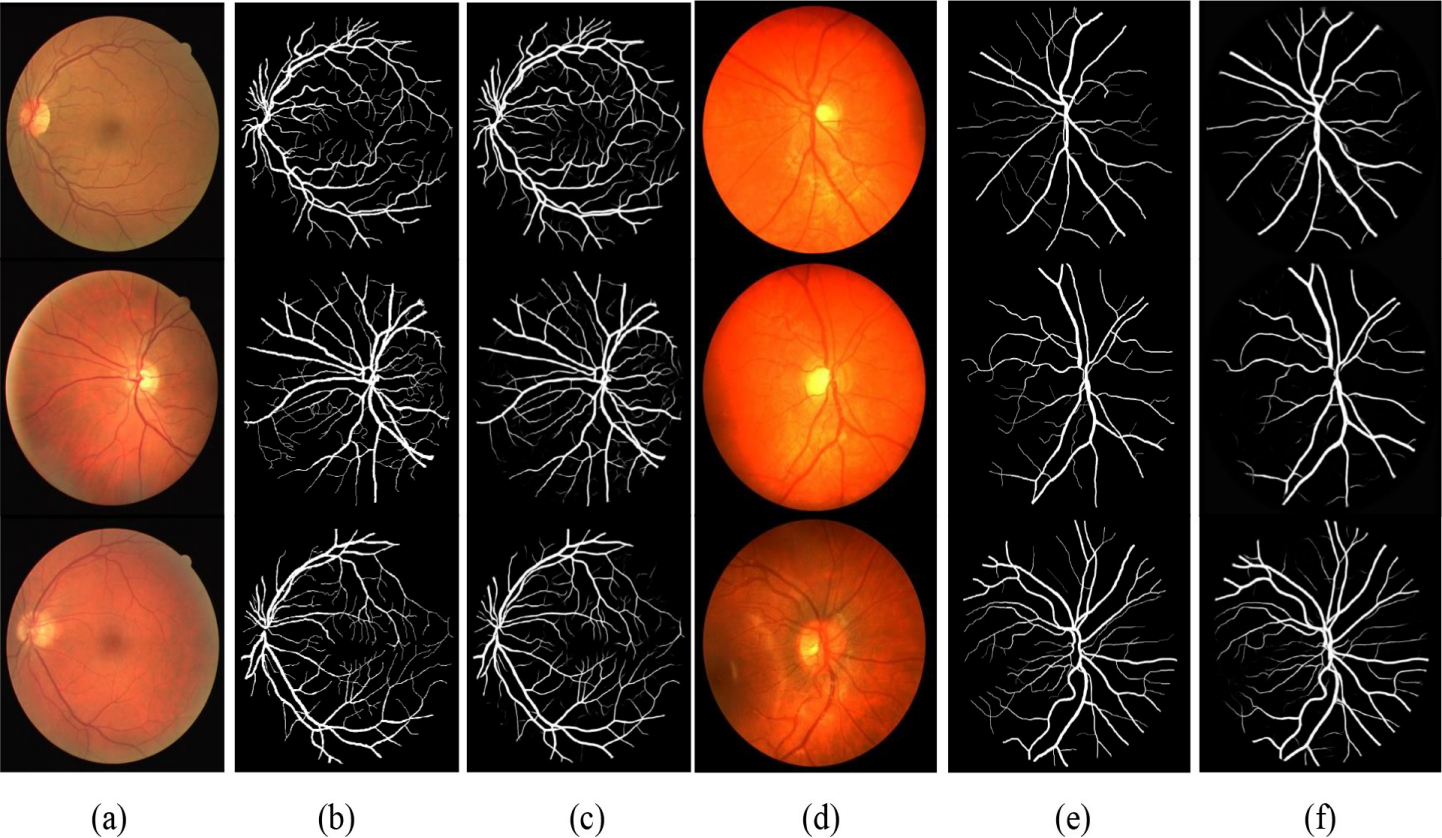
Some examples from two experiments on the DRIVE and CHASE DB1 datasets. The first three columns are from the DRIVE dataset. The last three columns are from the CHASE DB1 datasets. (a) and (d) Color fundus image, (b) and (e) ground truth, (c) and (f) segmentations.

Fig 3 shows some examples from our experiments. From the segmentation results of the two datasets, our results are very close to the gold standard, especially for some small blood vessels. We also verify the influence of different blocks on the results. According to the results in Table 1, our method is superior to the previous best in some key parameters. The highest ACC (0.9686%/0.9746%), the highest AUC(0.9842/0.9869), and the highest Se(0.8149/0.8420). That means residual convolution blocks and hybrid atrous convolution block are very useful for networks.

**Fig 4:**
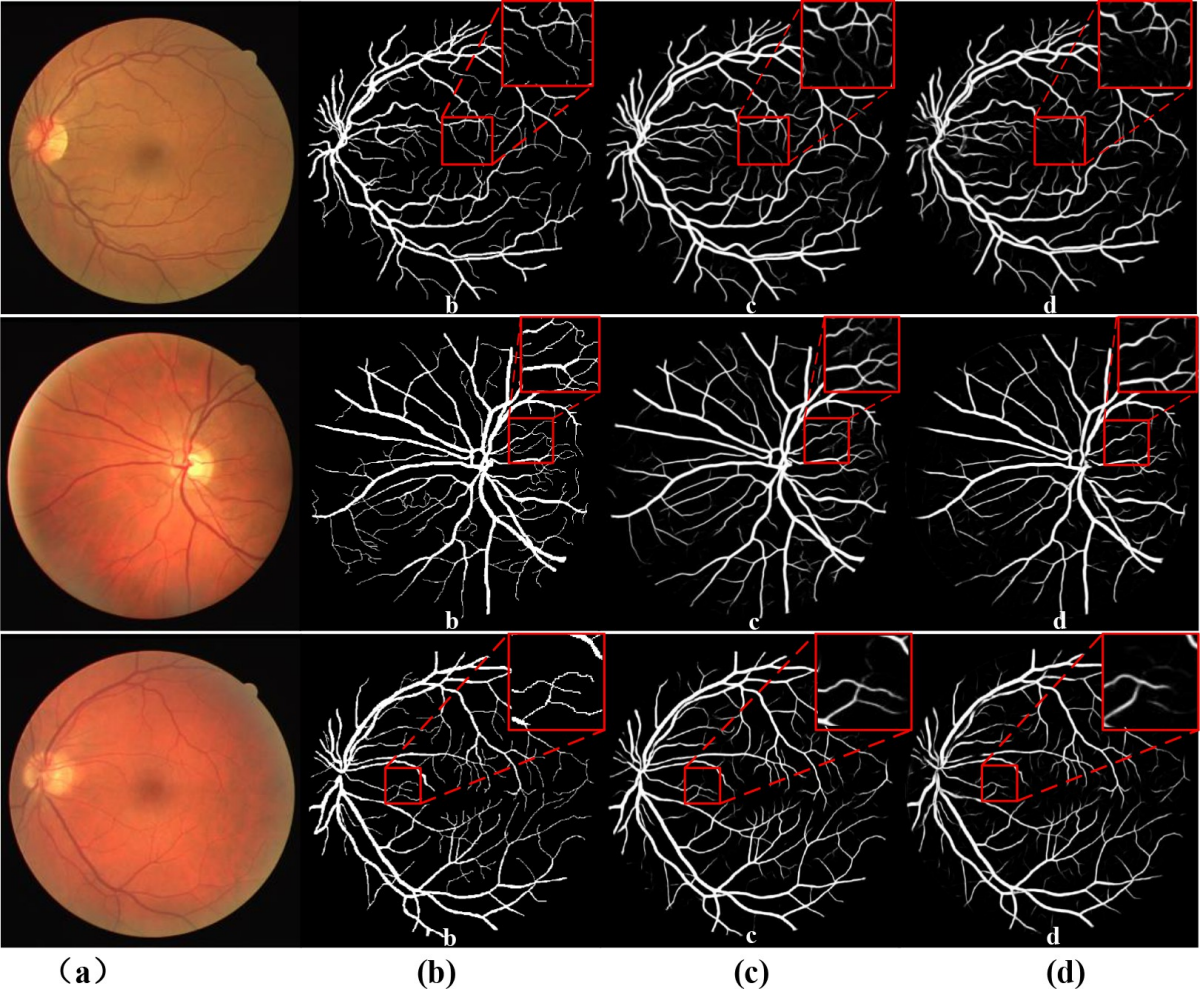
Comparison of ARU–Net and U–Net on DRIVE dataset: (a) color fundus images; (b) ground truth; (c) segmentation result by ARU–Net; (d) segmentation result by U–Net.

**Fig 5:**
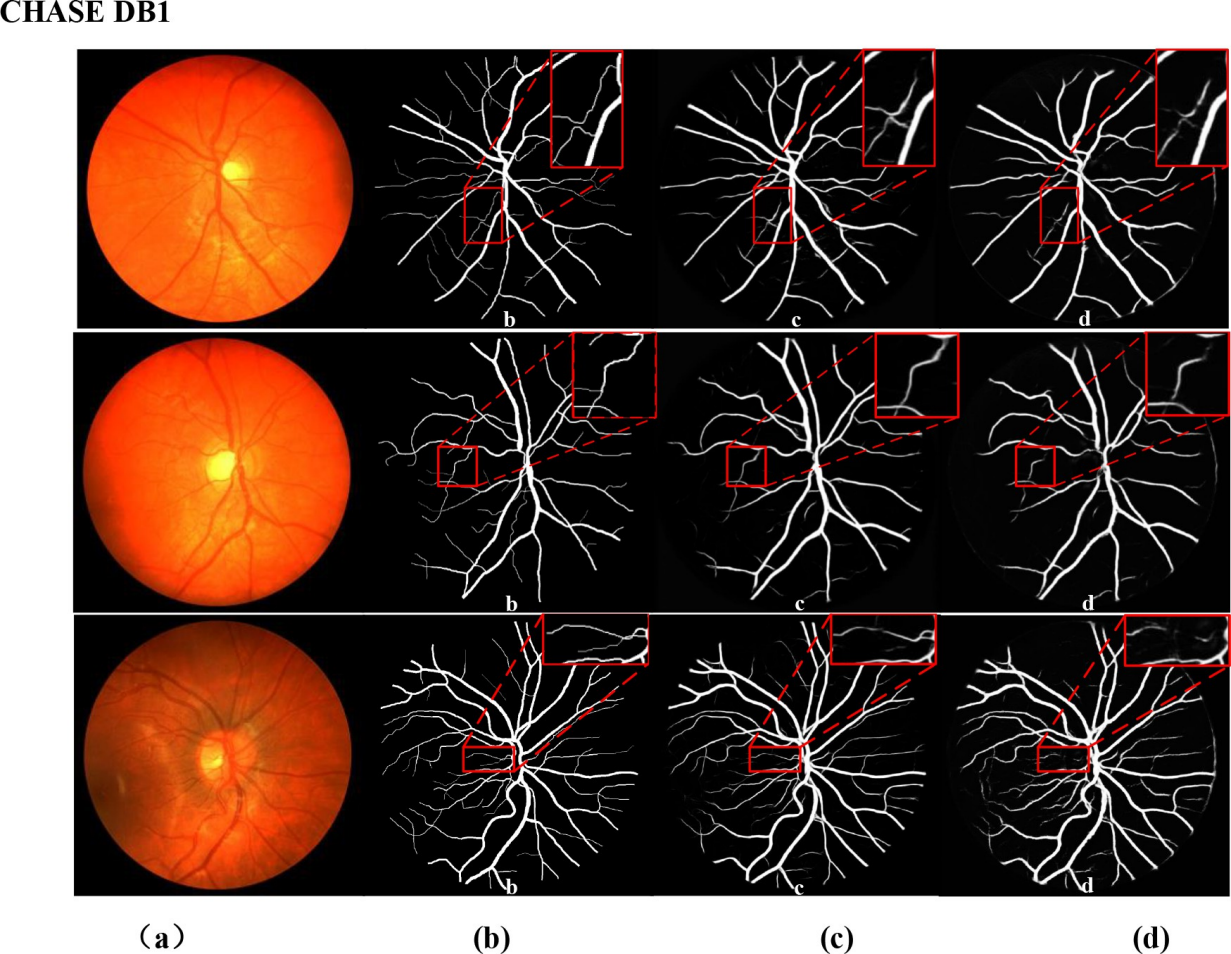
Comparison of ARU–Net and U–Net on CHASE DB1 dataset: (a) color fundus images; (b) ground truth; (c) segmentation result by ARU–Net; (d) segmentation result by U–Net.

The proposed method was also compared with U-Net in a segmentation experiment, and some examples of the results are shown in Figs 4 and 5. Fig 4 shows examples on the DRIVE dataset; Fig 4(A)–4(D) are the color fundus image, ground truth (manual annotation data), results by the proposed method, and results by U-Net. Fig 5 shows examples of the results on CHASE DB1. We can conclude from these results that our method can segment more vascular details, especially in capillary vessels (marked by a red box).

Finally, we compared ARU-Net with several state-of-the-art methods on the DRIVE and CHASE DB1 datasets, with results as shown in Table 4, which shows that ARU-Net performs best on both datasets as measured by Se, Sp, ACC, F1, and AUC. In details, on the DRIVE and CHASE DB1, our model has the highest AUC (0.21%/0.09% higher than the best before), the highest accuracy (1.08%/0.85% higher than the best before) and the highest sensitivity. F1 and specificity are generally comparable. Hence, our method achieves state-of-the-art performance for retinal vessel segmentation.

**Table 4:** Results of different methods on DRIVE and CHASE DB1.

	DRIVE					CHASE DB1				
Methods	Se	Sp	F1	ACC	AUC	Se	Sp	F1	ACC	AUC
Orujov [42]	**0.8380**	0.9570	**-**	0.9390	-	**0.8800**	0.9680	**-**	0.9500	-
Roychowdhury [43]	0.7395	0.9782	**-**	0.9494	0.9672	0.7615	0.9575	**-**	0.9467	0.9623
Azzopardi [44]	0.7655	0.9704	**-**	0.9442	0.9614	0.7584	0.9587	**-**	0.9387	0.9487
Jiong Zhang [45]	0.7743	0.9725	**-**	0.9476	0.9636	0.7626	0.9661	**-**	0.9452	0.9606
Fraz [46]	0.7406	0.9807	**-**	0.9480	0.9747	0.7224	0.9711	**-**	0.9469	0.9712
Li [47]	0.7569	0.9816	**-**	0.9527	0.9738	0.7507	0.9793	**-**	0.9581	0.9716
U-Net [31]	0.7537	0.9820	0.8142	0.9531	0.9755	0.8288	0.8288	0.7783	0.9578	0.9578
ResU-Net [32]	0.7726	0.7726	0.8149	0.9553	0.9553	0.7726	0.7726	0.7800	0.9553	0.9553
Orlando et. al. [48]	0.7897	0.9684	0.7857	0.9454	0.9506	0.7277	0.9712	0.7332	0.9458	0.9524
Yan et al. [49]	0.7653	0.9818	-	0.9542	0.9752	0.7633	0.9809	-	0.9610	0.9781
R2U-Net [50]	0.7799	0.9813	0.8171	0.9556	0.9784	0.7756	0.7756	0.7928	0.9634	0.9634
LadderNet [51]	0.7856	0.9810	0.8202	0.9561	0.9793	0.7978	0.9818	0.8031	0.9656	0.9839
RU-Net [52]	0.7751	0.7816	0.8155	0.9556	0.9782	0.7459	0.9836	0.7810	0.9622	0.9803
DEU-Net [53]	0.7940	0.9816	**0.8270**	0.9567	0.9772	0.8074	0.9821	**0.8037**	0.9661	0.9860
Vessel-Net [54]	0.8038	0.9802	**-**	0.9578	0.9821	0.8132	0.9814	**-**	0.9661	0.9860
Ours	0.8043	**0.9844**	0.8179	**0.9686**	**0.9842**	0.8099	**0.9856**	0.8005	**0.9746**	**0.9869**

In addition, adding multi-scale strategy can improve network performance, just like on the Drive dataset. The Se, Acc and AUC were improved (Se0.7772, ACC0.9553, AUC0.9759 respectively) [55,56]. In their study, an improved cross entropy loss function is applied, and uses CRFs as a post-processing strategy. The challenge is how to exploit the relationships between images at different scales. The segmentation result is greatly affected by the weight coefficient, which needs to be set manually. And compared with some other methods, the performance improvement is not obvious. For convolution neural network method with reinforcement sample learning strategy proposed by Guo et al. [57], Sp and ACC value was the lowest, and the final segmentation result was the worst. For U-net based on patch-based learning strategy, Se, Sp, ACC and AUC value were not the highest; however, the segmentation result was the best in the comprehensive evaluation.

## Discussion and conclusion

In medical image segmentation, common methods of data augmentation include random slice, rotation and mirror image, etc [58,59]. In general, the accuracy of the model on the training set is significantly increased, but in the validation set, the accuracy is not significantly improved, that is to say, the generalization ability of the model is not substantially improved. In order to improve the overall performance of the network, such as Se, Sp, ACC, AUC, and AUC, it is necessary to adjust the structure of the network, change the supervision function and the optimizer.

Unlike other data augmentation approaches, our method operates on the entire image. This has proven to be beneficial as our model is faster than the above methods. In addition, our model is able to obtain more natural and continuous segmentation masks and capture more detailed features. Furthermore, the introduction of hybrid atrous convolution blocks and modified residual blocks in our framework made it possible to utilize image at multiple scales, with corresponding oversight at each scale, helping our model to efficiently aggregate the outputs of different stages.

We presented ARU-Net, a segmentation structure to which atrous and residual convolution were added. This unit enlarges the receptive field without losing resolution. We replaced ReLU with LeakyReLU in the downsampling process. We evaluated the method on the DRIVE and CHASE DB1 benchmark datasets, and accuracy and sensitivity metrics demonstrated that our model can segment fundus vessels better than other models. The branches of many vessels, including very small vessels, were correctly segmented. Fundus diseases often reflect changes in the small shape of blood vessels.

**Fig 6:**
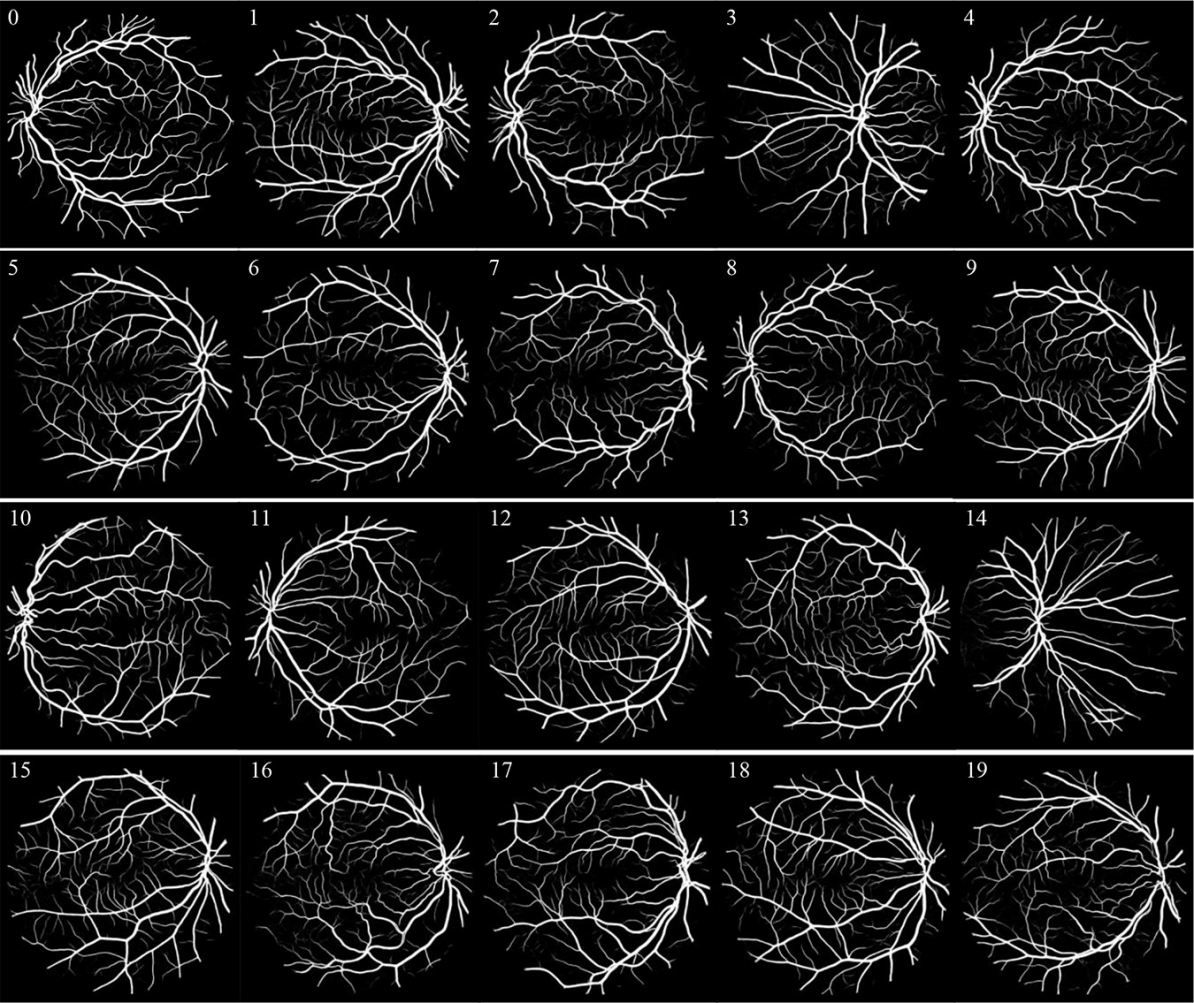
DRIVE datastes segmentation results.

**Fig 7:**
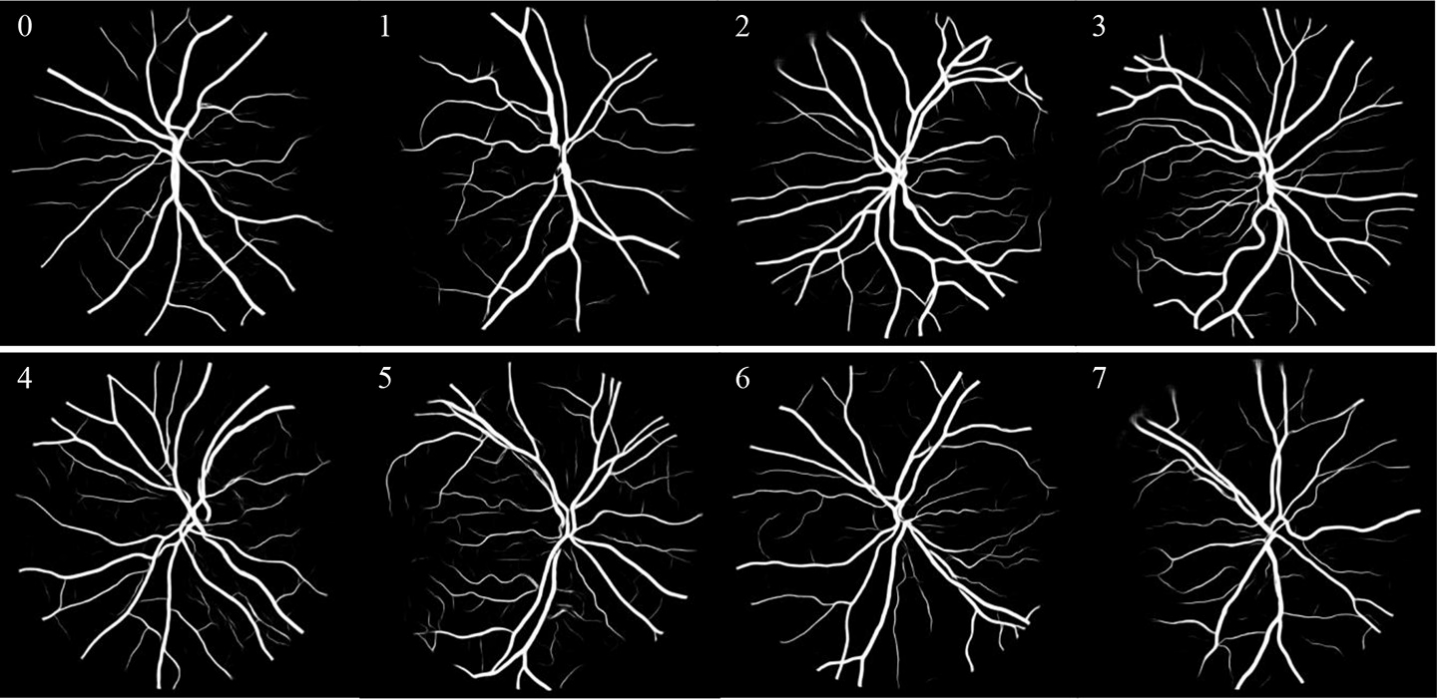
CHASE DB1 datastes segmentation results.

Therefore, the above methods can help doctors to diagnose diseases. However, the segmentation rupture of blood vessels can still occur in images with lesions. In the future, we’ll continue to explore how to ameliorate the problem of broken blood vessels, so that the segmentation results are closer to the real.The experimental results on the DRIVE and CHASE DB1 datasets are shown in Figs 6 and 7.
